# Nanomaterials for the Diagnosis and Treatment of Head and Neck Cancers: A Review

**DOI:** 10.3390/ma14133706

**Published:** 2021-07-02

**Authors:** Gustavo Ruiz-Pulido, Dora I. Medina, Mahmood Barani, Abbas Rahdar, Ghasem Sargazi, Francesco Baino, Sadanand Pandey

**Affiliations:** 1 Tecnologico de Monterrey, School of Engineering and Sciences, Atizapan de Zaragoza 52926, Mexico; A01166117@itesm.mx; 2 Medical Mycology and Bacteriology Research Center, Kerman University of Medical Sciences, Kerman 76169-14115, Iran; mahmoodbarani7@gmail.com; 3 Department of Physics, Faculty of Science, University of Zabol, Zabol 538-98615, Iran; 4 Noncommunicable Diseases Research Center, Bam University of Medical Science, Bam 76617-71967, Iran; g.sargazi@gmail.com; 5 Department of Applied Science and Technology, Institute of Materials Physics and Engineering, Politecnico di Torino, 10129 Torino, Italy; 6 Department of Chemistry, College of Natural Science, Yeungnam University, 280 Daehak-Ro, Gyeongsan 38541, Korea; Sadanand.au@gmail.com; 7 Particulate Matter Research Center, Research Institute of Industrial Science & Technology (RIST), 187-12, Geumho-ro, Gwangyang-si 57801, Korea

**Keywords:** biomaterials, nanomaterials, nanoparticles, stimuli-responsive materials, cancer treatment

## Abstract

Head and neck cancer (HNC) is a category of cancers that typically arise from the nose-, mouth-, and throat-lining squamous cells. The later stage of HNC diagnosis significantly affects the patient’s survival rate. This makes it mandatory to diagnose this cancer with a suitable biomarker and imaging techniques at the earlier stages of growth. There are limitations to traditional technologies for early detection of HNC. Furthermore, the use of nanocarriers for delivering chemo-, radio-, and phototherapeutic drugs represents a promising approach for improving the outcome of HNC treatments. Several studies with nanostructures focus on the development of a targeted and sustained release of anticancer molecules with reduced side effects. Besides, nanovehicles could allow co-delivering of anticancer drugs for synergistic activity to counteract chemo- or radioresistance. Additionally, a new generation of smart nanomaterials with stimuli-responsive properties have been developed to distinguish between unique tumor conditions and healthy tissue. In this light, the present article reviews the mechanisms used by different nanostructures (metallic and metal oxide nanoparticles, polymeric nanoparticles, quantum dots, liposomes, nanomicelles, etc.) to improve cancer diagnosis and treatment, provides an up-to-date picture of the state of the art in this field, and highlights the major challenges for future improvements.

## 1. Introduction 

The human head is a highly evolved structure with several important functions. It houses and protects important sense organs such as eyes, nose, ears, tongue, and related structures [1]. Besides the regular arrangement of different components between the head and neck, diseases produced in these vital structures and organs may threaten the health of a person [2]. Head and neck cancer (HNC) comprises a group of various malignant tumors that grow in the throat, larynx, mouth, sinuses, and nose [3]. Head and neck cancers are among the most common worldwide cancers and are located in the sixth place in terms of importance. Approximately 630,000 new patients’ cases are diagnosed annually and 350,000 deaths are reported every year. HNC squamous cell carcinomas (HNSCCs), which arise from the mucosal surfaces of the oral cavity, oropharynx, and larynx, include 90% of head and neck cancers cases [4]. Incidence and anatomy distributions of HNSCC may depend on different geographical locations.

Pathophysiology of HNC is an important parameter for better understanding of cancer origin. Traditionally, tumors are classified by stage and anatomic site of origin. Patterns of tumor growth and invasion may vary predictably with the anatomic barriers or pathways that prevent or allow extension. Within the head and neck, these sites are classified based on established anatomic parameters. The upper aerodigestive tract is organized into the following six site categories: nasopharynx, oral cavity, oropharynx, hypopharynx, larynx, and trachea. Beyond the upper aerodigestive tract, the paranasal sinuses, skull base, salivary glands, endocrine glands, skin, ear, and temporal bones are other possible sites where primary HNSCCs may arise. While HNSCC has traditionally been categorized by its anatomic site of occurrence, other factors may also be important in determining prognosis. Over the years, several different types of HNSCC have been described. Some histopathologic findings have been shown to have prognostic significance. Certain tumor characteristics, including keratin production, level of differentiation, nuclear appearance, mitoses, and host factors, such as inflammation, desmoplastic reaction, patterns of invasion, and vascular invasion, have been described as adjuncts to clinical staging for predicting outcome [5].

It was reported that consumption of alcohol and tobacco can increase the risk of this type of cancer by up to 80% [6]. Due to this, India, Bangladesh, and Pakistan are among the highest-risk countries. In the northern regions of America and Europe, HNSCCs include 5–10% of all new cancer cases. In the United States, 53,600 patients are diagnosed yearly and 11,500 deaths are recorded annually as associated with these types of cancer [7,8].

Most of HNCs can be developed in the flat squamous cells that create a thin layer of tissue on the surface of the head and neck. There are many ways for HNC treatment. The management of patients with this kind of cancer depends on the extent of the disease at detection. So, the main methods for the treatment of HNC are surgery, radiation, chemotherapy, and antibody-blocking therapy [9,10,11]. Surgical methods are the standard route for patients diagnosed with early-stage disease. The majority of patients, however, face advanced-stage disease that precludes surgery.

Most cancers that involve a tumor are staged in five broad groups. These are usually referred to with Roman numerals. They all tell you how advanced the cancer is. It is important to determine the stage of cancer and the resectability of the tumors for a better treatment approach. In many clinical outcome studies, stage remains one of the only valuable prognostic parameters. Stage 0 means there is no cancer, only abnormal cells with the potential to become cancer. This is also called carcinoma in situ. Stage I means the cancer is small and only in one area. This is also called early-stage cancer. Stage II and III mean the cancer is larger and has grown into nearby tissues or lymph nodes. Stage IV means the cancer has spread to other parts of the body. It is also called advanced or metastatic cancer. The primary and early-stage cancers, i.e., lesion stage (as T1 or T2 with no nodal involvement), are classified according to size and location relative to important surrounding structures and are best treated with surgery or radiation depending on the subsite and the expertise of the clinical team. Intermediate-stage tumors, i.e., infiltrative tumors, poor-prognosis T2 tumors, or exophytic T3 N0–N1 tumors, may benefit from a combined-modality approach. The locally advanced tumors are the unfavorable infiltrative T3 or T4 primary tumors with N2 or N3 lymphadenopathy. Patients with locally advanced tumors are best treated with concurrent chemoradiation if the tumor is unresectable, or if it is resectable but organ preservation is desired, or else if patients are receiving postoperative adjuvant radiation with concurrent cisplatin [5,12].

The molecular structures (receptors) and genetic change (biomarkers) that happen in head and neck cancer have allowed the detection of candidate routes for effective “targeted” approaches to therapy. Advances in the understanding of the molecular basis of HNC should help in the identification of new markers that could be used for the diagnosis, prognosis and treatment of the disease. Cancer is a genetic disease but does not imply inheritance; rather, the agents that bring about malignant transformation of a cell in the foundational step of tumorigenesis do so by inducing change in the tumor DNA. This may be by alteration in the base sequence (through mutation, deletion, insertion, or rearrangement), change in copy number of a chromosomal segment (through duplication, larger segment deletion, and loss of heterozygosity), alterations in the level at which a gene is transcribed through rearrangements that bring the gene into new association with promoter regions, or through epigenetic events including (hypermethylation of promoter regions) that block expression of mRNA into protein [13,14].

Multiple signaling pathways involved in the invasion process are influenced by genetic alterations in the development of head and neck cancer. Furthermore, several drugs have been tested to affect each of these pathways [15]. Conventionally available methods for HNC treatment suffer some major restrictions. The mainstays of medical imaging for detection of HNC patients are magnetic resonance imaging (MRI), computed tomography (CT), and positron emission tomography (PET). These conventional techniques suffer from relatively poor resolution and cannot recognize details of molecular changes. Furthermore, interpretation of imaging results can be complicated by difficult anatomy, edema or inflammation, scarring from prior treatment, and loss of detail changes because of patient’s movement [16]. So, the conventional methods for treatment of HNC have to face serious challenges.

**Figure 1:**
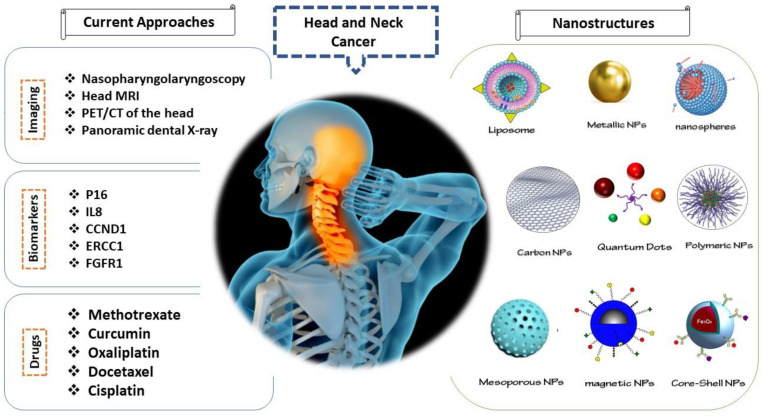
Schematic representation of current approaches for diagnosis and treatment of head and neck cancer and use of nanostructures to enhance efficiency of these methods. Note: CT: Computed tomography, PET: positron emission tomography, MRI: magnetic resonance imaging, ERCC1: excision repair cross complementing group 1, p16: cyclin-dependent kinase inhibitor 2A, IL8: interleukin-8, CCND1: cyclin D1, FGFR1: epidermal growth factor receptor group 1, NPs: nanoparticles.

Nanotechnology is expected to develop a wide range of instruments for detection and treatment of sickness in medicine [17,18,19,20,21,22]. Nanostructures with small particle size distributions are well applied to interact with biological molecules and diverse structures developed inside living cells [23,24,25,26]. Most nanoparticles can be used as a nanofluid typically made of metals, oxides, carbides, or carbon nanotubes [27,28,29,30]. Nanostructures present the appropriate size range for imaging and manipulation at the molecular level. The capacity to effectively control the nanosized nature of surface chemistry allows interacting at molecular levels [31]. Figure 1 displays the application of some nanostructured devices for the detection and treatment of HNC.

Nanoporous materials also have appealing properties, including uniform pore morphology, high surface area, and small particle size distribution, and have been applied in different areas, such as medical applications [32,33,34,35,36]. The physicochemical properties of these nanomaterials can be potentially suitable in detection and treatment of HNC [37]. Just to provide some examples, magnetic nanostructures were widely utilized due to their unique features such as magnetic susceptibility and stability. Li et al. developed methotrexate-conjugated and hyperbranched polyglycerol-grafted Fe_3_O_4_ magnetic nanoparticles for targeted anticancer effects in HNC. The methotrexate acts as an efficient cancer-targeting ligand and antitumor drug [38]. Poláková et al. used fibrous nanostructures with high physicochemical properties for cancer detection and treatment by local anticancer therapy. This research developed a new option for treatment of the HNC due to selectivity in diverse configurations of the nanofibrous structures [39].

## 2. The Need for Nanomaterials for Treatment of Head and Neck Tumors and Cancers

Currently, the most common treatments for HNC are chemotherapy and radiotherapy, which often fail to eradicate tumors because they lack specificity to differentiate cancer cells from healthy proliferating cells [40]. Chemotherapy presents a scattered distribution, restricting its effectiveness and leading to severe side effects [41]. Radiotherapy commonly develops tumor resistance, resulting in poor outcomes and negative prognoses [42].

Nanomedicine has emerged as a new technology for improving chemo- and radiotherapies through the use of nanoparticle drug carriers to develop tumor-targeting drugs [43], which will improve the efficacy and safety of cancer therapies. Nanoparticles (NPs) are synthetic particles ranging between 1 and 100 nm in diameter that are commonly classified into metallic NPs, metal oxide NPs, mesoporous NPs, polymeric NPs, nanomicelles, and liposomes, as Figure 1 shows [40,41,44,45].

Nanocarriers smaller than 100 nm represent an optimal vehicle for systemic administration due to their prolonged circulation in blood [46]. This small size promotes a proper uptake of nanoparticles by tumoral cells via enhanced permeability and retention (EPR) effect, producing local accumulation and cytotoxic effect on those cells [47].

Otherwise, an ideal nanoparticulate system for drug delivery should exhibit the following characteristics: (1) high drug bioavailability, (2) specific targeting, (3) controlled release, (4) low immunogenicity, (5) ability to deliver poorly traditionally bioavailable agents, and (6) sufficient loading capacity [48]. Thus, the review describes and compares (Table 1) the main characteristics of the most used nanosystems for delivering anticancer drugs during HNC treatments. A schematic representation of current approaches for diagnosis and treatment of head and neck cancer and the use of nanostructures to enhance the efficiency of these methods is depicted in Figure 1.

## 3. Metallic and Metal Oxide Nanoparticles

Different metallic and metal oxide nanoparticles with a size of 1–100 nm have emerged as potential “weapons” for treating HNC, including cerium oxide, gadolinium, gold, iron oxide, and silver, which are synthetized mainly for amplifying radiation effects [43,49,50] due to their high X-ray absorption and ability to emit secondary energy in form of photoelectrons, auger electrons, and X-rays into the surrounding tissue [42,51,52,53]. Moreover, metallic and metal oxide nanoparticles provide a versatile platform for surface modification through covalent bonds, complexation, or coupling to the capping agents [54,55,56,57].

### 3.1. Gold Nanoparticles

Gold (Au) nanoparticles exhibit two main mechanisms for treating HNC: photothermal therapies of malignant tumors and radiosensitization of the cancer cells [58], based on their unique physicochemical properties, including biocompatibility, preferential accumulation in tumors, photostability, photothermal conversion, and optical and multifunctionalization features [59]. 

Photothermal ablation is a mechanism for treating malignant tumors by using a photothermal agent to produce an intense and highly localized hyperthermic effect [60]. Au NPs exhibit unique surface plasmon resonance (SPR) properties, in which gold electrons resonate in response to incoming radiation, promoting the absorption and light scattering to obtain localized heating [50]. This efficient conversion of light is achieved at the near-infrared (NIR) wavelength range, 700–1000 nm, and is suitable because the electron–photon interaction at this wavelength presents low scattering and low absorption by blood and soft tissues [61,62]. Meanwhile, it produces apoptosis of cancer cells as a consequence of raising the temperature of the tumor to around 45 °C or more, which is considerably above the body physiological temperature (36–37 °C), leading to severe alterations in the tumor cell membrane, inhibition of DNA synthesis, and destruction of the cytoskeleton [59,63,64]. It is worth highlighting that gold nanorods or gold nanostars are more convenient for photothermal therapies based on their higher efficiency in the absorption of near-infrared light [65].

On the other hand, Au NPs are very attractive radiosensitizers that intensify the radiation dosage through a strong X-ray absorption followed by the emission of secondary electrons [66] which stimulates the radiolysis of intracellular water, a high production of free radicals, a radiation-induced reactive oxygen species (ROS) generation, oxidative stress, DNA damage, and interferences in the cell cycle that are associated with increased necrotic and apoptotic cell death [63,67], triggering cytotoxic effects in tumoral cells [58]. 

In addition, Au NPs can be coated with targeting ligands and surface-engineered with anticancer drugs for combining radiotherapy with chemotherapy [68], in order to improve the outcomes through a synergistic effect in which the nanoparticles act simultaneously as a radiosensitizer and as a targeted carrier of chemotherapeutic agents [42].

### 3.2. Gadolinium Nanoparticles

Gadolinium (Gd) is a lanthanide whose potential radioenhancing properties have been widely explored, despite the fact that, unlike other metallic nanoparticles, Gd NPs are not metal-core particles, thus they typically refer to Gd chelates or Gd-based nanoparticles [69]. Nonetheless, Gd NPs represent an interesting method for treating HNC through an efficient radiosensitizing activity in radioresistant cellular and animal models of head and neck squamous cell carcinoma [70]. Therefore, Gd chelates or Gd-based nanoparticles are used for enhancing radiation dose during radiotherapy by inducing the activation of an autophagy pathway when the tumoral cells are exposed to X-rays, which improves the effectiveness of radiotherapy while reducing collateral damage [71]. In the same way, the radiosensitizing properties of Gd NPs stimulate photocytotoxic effects through the production of extra ROS and water radiolysis products [72]. Furthermore, Gd NPs exhibit some advantages during HNC treatments such as low toxicity, enabled renal elimination, and preferential uptake in tumors through an EPR effect [43]. 

### 3.3. Iron Oxide Nanoparticles

Iron oxide (Fe_3_O_4_) nanoparticles have been approved by FDA as a photothermal agent for application in cancer treatments due to their broad absorption in the near-infrared (NIR) range [73]. Moreover, superparamagnetic Fe_3_O_4_ NPs can exhibit magnetic hyperthermia by converting an external high-frequency field energy into thermal energy, which produces thermal ablation when the temperature raises over 50 °C to cause irreversible cell damage, inhibition of tumor growth, and necrosis of tumoral cells in HNC [74,75]. Nonetheless, Fe_3_O_4_ NPs are easily recognized by the innate immune system as invaders, so they are rapidly cleared from the systemic circulation by the reticuloendothelial system (RES) [76]. Another main problem during cancer treatments is the low targeting properties of superparamagnetic Fe_3_O_4_ NPs, which results in lower efficacy and greater side effects [77].

Different types of coating and surface functionalization have been studied for countering the rapid clearance of Fe_3_O_4_ NPs and for stimulating their accumulation in cancer cells through the EPR effect [78]. The novel approaches for coating magnetic Fe_3_O_4_ NPs include cell-membrane coating and anti-CD44 antibodies, both with the purpose of targeting the overexpressed CD44 receptors in cancer stem cells and the evasion of immune system [73,77] to their specific cell killing potential without damaging the surrounding healthy tissue [74].

In addition, magnetic drug targeting has been explored for guiding and inducing the accumulation of superparamagnetic Fe_3_O_4_ NPs into a specific site (commonly tumors) by strong external magnetic field gradient [46,74]. This approach is mainly used when superparamagnetic Fe_3_O_4_ NPs are employed as nanocarriers by functionalizing their surface for delivering chemotherapeutic agents in order to increase the efficacy of cancer treatments [78]. However, superparamagnetic iron oxide NPs can also be crosslinked with different polymeric matrixes, such as cellulose nanocrystals, to form hierarchically organized networks similar to “nanocages” with the objective of capturing circulating tumor cells in the blood during HNC treatments [79].

### 3.4. Silver Nanoparticles

Silver (Ag) nanoparticles have demonstrated some antiproliferative properties against cancer cells by inducing cellular cytotoxicity in cancer cells through different mechanisms such as generating ROS and free radicals, genomic instability, DNA fragmentation, disruption of calcium (Ca^2+^) homeostasis, cytoskeletal weakening, damage of intracellular organelles, and interruption of some intracellular signal transduction pathways, which results in cancer cell apoptosis [80,81]. Therefore, Ag NPs show great potential for new therapeutic approaches by improving the sensitivity of current therapies [82]. Ag NPs can even act as cell sensitizers for photothermal therapy, whereas they provide different alternatives for delivery of chemotherapeutic drugs through their surface functionalization and conjugation in a clearly synergistic effect which can enhance the efficacy of the treatment [60]. Recent studies have demonstrated that Ag NPs ingestion is safe and could be associated with a complete and sustained regression of HNC, including metastases to other organs such as the liver or the lungs [83].

### 3.5. Cerium Oxide Nanoparticles

Cerium oxide (CeO_2_) nanoparticles have attracted a special interest due to their dual radioenhancing and radioprotective properties based on their redox-modulatory activities, which are related to an antioxidant/pro-oxidant reversal property that is useful for sensitizing cancer cells and protecting normal cells from ROS during radiation therapy [69,84]. This characteristic behavior of CeO_2_ NPs is related to the presence of the two-valence state of cerium and its ability to switch between oxidation states, resulting in a predominant superoxide dismutase or catalase behavior [85]. At neutral pH conditions, CeO_2_ NPs adopt an enzymatic defense mechanism similar to superoxide dismutase, catalase, peroxidase, and oxidase activities [86], in which CeO_2_ NPs mimic superoxide dismutase activity by converting Ce^3+^ to Ce^4+^ and diminishing the levels of superoxide and free radicals such as nitric oxide, hydroxyl, and hydrogen peroxide [87], followed by catalase activity stimulated by the reconversion of Ce^4+^ into Ce^3+^, in which the hydrogen peroxide is decomposed into water and hydrogen molecules [85]. Otherwise, the antioxidant activity of CeO_2_ NPs is significantly reduced at low pH, suggesting that under acidic environments of highly glycolytic tumors their catalase activity is reduced, inducing oxidative stress and cytotoxicity in cancer cells that results in apoptosis of cancer cells and inhibition of tumor metastasis [40].

## 4. Liposomes

Liposomes represent one of the most attractive and widely investigated nanocarriers for efficient delivery of anticancer agents in humans [88]. They are small artificial spherical vesicles formed by an amphiphilic phospholipid bilayer with an inner aqueous cavity, amenable for encapsulating molecules with different polarities [89,90]. Nanoliposomes are nanometric structures with the range of 20 to 150 nm during storage and applications. According to their structure and size, liposomes are divided into small unilamellar vesicles (SUV, 20–50 nm), medium unilamellar vesicles (MUV, >100 nm), large unilamellar vesicles (LUV, >560 nm), and multilamellar vesicles (MLV, 170–5000 nm) [91]. Hydrophobic drugs are entrapped inside the lipid bilayer membranes, while hydrophilic drugs are encapsulated in the aqueous core of the vesicles [92]. Liposome formulation is highly flexible, allowing the modification in the composition of its bilayer components to achieve the desired rigidity, permeability (or impermeability), and stability [93]. In addition, the presence of sterols, such as cholesterol, in liposome structure promotes membrane permeability due to its close resemblance to the mammalian cell membrane [94]. These properties provide several advantages to liposomes, such as biocompatibility of the phospholipid bilayer, cellular uptake via pinocytosis, high drug payload, drug protection against biodegradation during blood circulation, simple synthesis methods, low batch-to-batch variation, and easy surface conjugation to obtain specific functions and targeting [95,96], which allow enhanced effectiveness of different chemo- and radiotherapies through increased drug accumulation in tumor cells and enhanced biodistribution and pharmacokinetic parameters [97].

For instance, several clinical trials have confirmed that liposomes loaded with chemotherapeutic drugs, such as cisplatin, present an enhanced drug tolerability, a longer half-life, and a favorable safety profile in terms of reduced nephrotoxicity, neurotoxicity, leukopenia, neutropenia, nausea/vomiting, and asthenia during HNC treatment [98]. 

**Figure 2:**
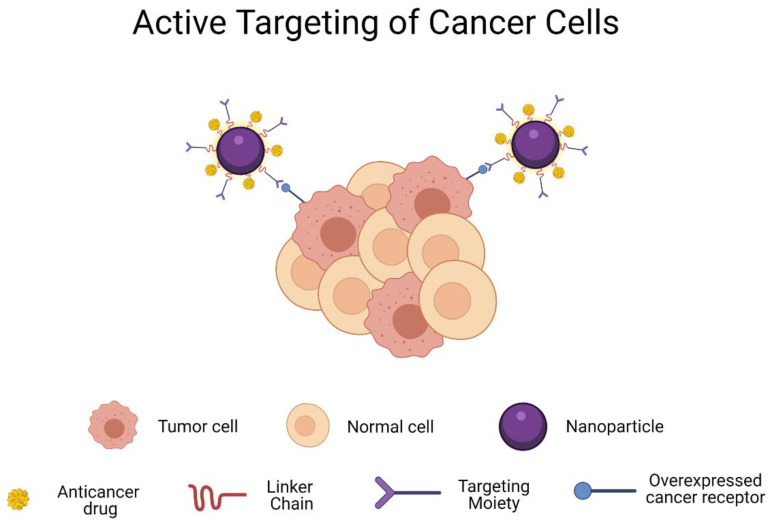
Schematic representation of active targeting of cancer cells by targeting moieties. Targeting moieties allow a specific binding with the overexpressed receptors on tumor cells. Created with BioRender.com.

Additionally, the liposome surface can be conjugated with different peptides to target specific receptors that are overexpressed in tumoral cells, such as Hsp47/CBP2 receptor in the case of HNC [99]. Immunoliposomes could be developed by conjugating the liposomes with antibodies for active targeting (as Figure 2 shows) of overexpressed signals in HNC cells, such as epidermal growth factor receptors (EGFRs) [96].

Otherwise, liposomes can be coated with polymers such as polyethylene glycol (PEG) for acquiring an even longer blood circulation by decreasing the level of RES-mediated plasma clearance, while simultaneously they can be conjugated with ligands for interacting with specific extracellular domains of HNC cancer cells [100]. However, a prolonged therapy of PEGylated liposomes loaded with particular chemotherapeutic drugs, such as doxorubicin, could be associated with an increased risk of secondary HNC due to a high cumulative dose of a compound with very aggressive side effects [101]. Instead, the administration of multiple doses of radiopharmaceuticals with PEGylated liposomes has shown an improved therapeutic effect by tumor accumulation with no acute toxicity detected [102]. 

A new method of delivering hydrophilic chemotherapeutic drugs loaded inside liposomes has recently emerged to increase their internalization in tumoral cells through creating some membrane defects by short pulses of strong electric fields, in a technique called electroporation. This methodology tends by itself to kill the cancer cells closest to the electrodes, but it can also enhance drug release by altering the lipid layer of the liposome, which results in a more powerful treatment [103]. Another interesting approach relies on using matrix liposomal platforms for delivering radiotherapeutic drugs: for example, a metalloproteinase (MMP)-sensitive liposomal matrix could ensure a proper bioavailability of a radiosensitizer at the tumor site by converting the PEGylated anionic liposomes into a dePEGylated cationic liposomes due to the high local expression of MMPs at tumor microenvironment, leading to an easy internalization of MMP liposomes by cancer cells [104]. Similarly, magnetic liposomes can be developed for transporting active molecules and hyperthermal drugs based on their magnetic core which is oriented by an external magnetic field. The most common magnetic core is iron oxide, which can simultaneously act as a radiosensitizer with minimal toxicity [95].

## 5. Nanomicelles and Microemulsions

Nanomicelles represent novel colloidal structures around 5–100 nm in size that are prepared with amphiphilic monomers that self-aggregate to provide high encapsulation efficiency and drug loading capacity [105]. They consist of two main sections: (1) hydrophobic (nonpolar) tails that form the “inner core”, which traps and stabilizes the encapsulated drug, and (2) hydrophilic (polar) heads that form the “outer shell”, which controls the pharmacokinetic properties, drug targeting, and specificity [106]. Although nanomicelles exhibit a very similar amphiphilic structure to liposomes, there are a couple of specific differences between them: (1) liposomes are composed of a lipid bilayer that surrounds an aqueous internal compartment, while micelles are a lipid monolayer with a fatty acid core and polar surface, and (2) liposomes are composed with a high concentration of amphiphilic molecules, whereas nanomicelles present low concentration of amphiphilic molecules [107].

Nanomicelles exhibit promising opportunities for drug delivery based on their properties to encapsulate hydrophobic drugs in the inner core, allowing an efficient delivery of poorly soluble drugs, while the outer core can be easily modified by conjugating hydrophilic drugs or specific ligands for specific targeting or co-delivery [108]. Thus, nanomicelles can act as a protective shell for hydrophobic drugs by reducing their contact with the environment, which results in a lower degradation and improved bioavailability with reduced side effects [109]. In addition, nanomicelles are suitable for drug accumulation in solid tumors by the EPR effect, due to their significant stability during circulation time, allowing them to carry more drug to the tumor by extravasation through capillary discontinuities generated by tumor angiogenesis [110].

Otherwise, nanomicelles have been recently studied as a vehicle for chemotherapeutic drugs, such as cisplatin, demonstrating tumor growth inhibition and prolonged survival in patients in HNC cancer treatment, because the micelles minimize the degradation caused by glutathione (a molecule that is abundantly produced in cancer stem cells) and allow a delayed drug release [111]. Moreover, curcumin delivery by nanomicelles has been tested for managing the oral mucositis associated with HNC, and the results demonstrate that curcumin-entrapped nanomicelles exhibit a higher oral bioavailability (by countering its rapid metabolization), an enhanced systematic exposure, and no toxicity or side effects [112]. In addition, metallic compounds such as ruthenium(II)–arene complexes can be conjugated with nanomicelles to counteract their poor cellular internalization [113]. However, some drugs require repeated doses to carry out a proper concentration inside the tumor during HNC treatments. Thus, nanomicelles represent an alternative by improving chemotherapeutics’ half-life and inducing higher drug accumulation at the targeted site, resulting in an enhanced antitumor effect [110].

Nanomicelles can also be used for novel HNC treatment approaches that consider the co-delivery of chemotherapeutic drugs for increasing their antitumor effectivity by exhibiting stronger cytotoxic effects towards cancer cells compared to free drugs [114]. For instance, Zhu et al. investigated the mixture of methotrexate (MTX) and salinomycin (SAL), in which MTX was conjugated in the hydrophilic outer shell due to its affinity for the overexpressed folate acid receptors in tumor cells, while SAL was encapsulated in the hydrophobic inner core to be released in the central regions of solid tumor [115]. Nevertheless, nanomicelles present some limitations for drug delivery uses, such as premature drug release, poor control of sustained release, and inability to encapsulate hydrophilic agents [105].

In the same way, microemulsion-based formulations represent a promising class of drug-delivery nanovehicles for oral and parenteral administration by encapsulating poorly soluble and poorly bioavailable chemotherapeutic drugs [114]. Microemulsions prepared with appropriate surfactants usually exhibit an enhanced colloidal stability in order to prevent nanoparticle aggregation and to reduce side effects impact in healthy tissue. A clear example is the emulsion of Pluronic–MTX conjugated nanomicelles for treating cancer [116].

## 6. Polymeric Nanoparticles

Polymeric nanoparticles are colloidal particles with a mean diameter between 100 and 300 nm that are prepared with biocompatible polymers for controlled and targeted transport of drugs [106,117]. They represent a great and versatile platform for improving HNC therapy due to their high encapsulation efficiency of hydrophobic anticancer drugs, which usually exhibit poor pharmacokinetics and inappropriate biodistribution [118]. Furthermore, polymeric nanoparticles promote the accumulation of encapsulated drugs inside tumor tissues through the EPR effect [119,120] with a subsequent controlled release to increase its antitumoral efficacy [121,122]. In the same way, polymeric nanoparticles can be loaded with radiosensitizing drugs for countering radiotherapy resistance and reducing side effects [123]. Meanwhile, hydrophilic drugs are usually embedded in the nanoparticle surface [54].

Additionally, polymeric nanoparticles allow the delivery of a combination of therapeutic agents with a reduced intensity of side effects. The surface of polymeric nanoparticles can be modified for triggering an efficient cell-membrane penetration and cellular internalization in the acidic environment of tumoral cells, as compared to the neutral pH of healthy cells [124].

Otherwise, polymeric nanoparticles are divided into nanocapsules and nanospheres, based on their morphology. A nanocapsule refers to a polymeric shell that surrounds an aqueous or oily core in which the active compound is confined (usually dissolved), whereas nanospheres are a polymeric network in which the active compound and the polymer are uniformly dispersed [117,125].

Natural (e.g., chitosan and hyaluronic acid) and synthetic (e.g., poly(lactide-co-glycolide) and polyethylene glycol) polymers have been studied for administering chemotherapeutic drugs and radiosensitizers based on their biocompatibility and biodegradability [54,106]. After drug release, the polymeric matrix is usually degraded into innocuous molecules such as water and hydrogen- and nitrogen-containing molecules, which are excreted from the body [126].

However, natural polymeric systems present variability between batches, irregular release kinetics, and mild immunogenicity, which sometimes restrict their use as vehicles for anticancer molecules [54] in comparison with synthetic polymers that offer the additional advantages of high purity, reproducibility, and well-known chemical composition [127]. 

### 6.1. Chitosan Nanoparticles

Chitosan is a natural cationic polymer that exhibits high biocompatibility, biodegradability, reduced toxicity, low cost of preparation, high encapsulation rate, controllable drug release kinetics, and targeting properties at specific tissues [128]. In addition, chitosan NPs provide drug stability, reduce adverse reactions, present facilitated transmucosal drug delivery, and have mucoadhesive features, which make them appropriate nanocarriers for delivering entrapped chemotherapeutic agents during HNC treatments [129]. For instance, oxaliplatin (OXPt) has been successfully incorporated in chitosan NPs to interact with the mucosa for a prolonged time, leading to an initial drug burst effect followed by a long-term sustained release with the opportunity to accumulate in the tumor tissue in a more concentrated way, thus increasing the rate of apoptosis in oral tumors [130]. Moreover, the controlled release from chitosan nanovehicles is associated with reduced kidney toxicity and decreased inflammatory response, without affecting their anticancer activity [131].

In the same way, chitosan has a pKa value around 6.5, which plays a key role in its drug-releasing and mucoadhesive properties, with a positive charge at low pH due to amino group protonation that allows interaction with negatively charged components of mucus [127]. Therefore, chitosan NPs can be engineered to develop a pH-sensitive tumor-targeting property under the acidic conditions of the tumor microenvironment, followed by an enhanced mucoadhesive activity that stimulates NP internalization by endocytosis to reduce chemotherapy-induced damage in healthy tissues [128]. For that reason, chitosan NPs are used in novel mucoadhesive topical formulations to deliver anticancer agents through iontophoresis, in which a small electric current is applied to transport hydrophilic anticancer molecules (i.e., drug-loaded chitosan NPs) for treating oral tumors through rapid penetration of the NPs into the mucosa [130].

Otherwise, the functional groups on the surface of chitosan, i.e., hydroxyl (-OH) and amine (-NH_2_), are highly reactive, allowing the easy modification of their surface through different chemical reactions [127], which make it an appropriate compound to administer drugs such as cisplatin, by coordinating bonds between the carboxylic moieties available in the polymer backbone and the center of the drug [131]. Moreover, specialized ligands can be conjugated with chitosan functional groups for interacting with specific cell surface receptors leading to NP endocytosis. The most common targeted receptors in cancer cells are the folate receptor, CD44 receptor, EGFR, low-density lipoprotein receptors, and integrins [129].

### 6.2. Hyaluronic Acid

Hyaluronic acid (HA) is a natural glycosaminoglycan polysaccharide that has been extensively studied as a safe carrier or coating material for the delivery of anticancer agents, based on its unique properties [132]. Since HA represents an extracellular matrix constituent of connective tissues, it exhibits biodegradability, biocompatibility, nonimmunogenic properties, and specific binding ability with the overexpressed surface receptors in tumoral cells: CD44 and receptor for hyaluronan mediated motility (RHAMM) [133,134]. 

However, HA has some disadvantages such as poor stability and easy degradation, which can be countered with an appropriate chemical modification or conjugation with some therapeutic molecules [135]. For these reasons, HA is not commonly used alone for preparing nanoparticles, but it can be applied as a protective layer with the capability of improving drug pharmacokinetic properties [136]. Nevertheless, HA conjugation has been demonstrated to increase drug solubility, stability under biological conditions, a prolonged time in blood circulation that leads to a higher passive targeting, and a specific affinity for some overexpressed cellular receptors [137]. Therefore, HA is usually conjugated with hydrophobic and amphiphilic chemotherapeutical drugs that are required to overcome multidrug resistance (MDR) by actively targeting CD44 receptors (as Figure 2 illustrates), facilitating antitumor drug entrance into the tumor cells via receptor-mediated endocytosis with reduced toxic side effects and an enhanced efficacy at lower doses [138]. For example, HA–cisplatin nanoconjugates have been developed for local therapies of HNC, in which the conjugate stabilizes the cisplatin in the bloodstream and targets CD44 receptors of tumoral cells, followed by an accumulation of drug at tumor area based on an EPR effect to improve their therapeutic effect [133].

On the other hand, HA can be conjugated on the surface of metallic NPs through covalent amide bonds to improve their targeting and stability properties with the purpose of maximizing their anticancer effects in radio- and phototherapies. For instance, HA-coated Fe_3_O_4_ NPs represent a promising multifunctional platform for magnetic hyperthermia therapy due to their good colloidal stability, biocompatibility, high heating efficacy, and specific interaction with overexpressed receptors in HNC cells [134]. Furthermore, HA offers interesting coating features for mesoporous NPs, such as mesoporous silica, in three main aspects: (1) acting as a barrier that prevents drug release through the NP pores, (2) protecting drugs from the harsh conditions in the bloodstream, and (3) targeting specific receptors to promote an effective internalization of mesoporous NPs [136].

### 6.3. Poly(lactic-co-glycolic Acid) Nanoparticles

Poly(lactic-co-glycolic acid) (PLGA) is a biocompatible copolymer approved by the Food and Drug Administration (FDA) and the European Medicine Agency (EMA) and is widely used in the fabrication of nanoparticles for encapsulating and enhancing the properties of hydrophobic chemotherapeutic drugs, which results in a controlled drug release, lower dosage requirement, and reduced side effects [139]. PLGA properties can be tuned by modifying the molecular weight and changing the ratio of lactic to glycolic acid, in order to control the release rate of a drug or the biodegradability of the nanoparticle [47]. Another significant advantage of PLGA NPs as drug delivery vehicles is their property of being easily endocytosed by tumor cells, where they are transported into acidic endolysosomal compartments. When PLGA NPs are exposed to an acidified environment, the nanoparticles undergo degradation and decomposition by the hydrolysis of PLGA into lactic and glycolic acid monomers, releasing the antitumoral drug [140,141]. Moreover, the diameter of PLGA NPs of around 50–800 nm can improve the effectiveness of the delivery, because the vessels of healthy tissues have a space of 15–30 nm between cells, while inflamed regions present edema and consequently wider spaces between cells [139]. These attractive features of PLGA-based NPs make them promising delivery vehicles for chemotherapeutic drugs such as docetaxel (DTX), which has shown a localized in situ delivery to the tumor site and an increased antiproliferative efficiency compared with free DTX in a dose-dependent manner [142].

Furthermore, PLGA NPs have been developed for the successful encapsulation and administration of photosensitizers, considering that most of them are hydrophobic and need to maintain their stability for a prolonged circulation in the blood in order to reach an appropriate accumulation in tumor tissues. One good example is the encapsulation of pheophorbide (Pba), which has shown stability after one week, prolonged blood circulation, and a faster uptake on cancer cell lines, with an effective killing of tumoral cells in mice by photodynamic effect [143]. However, PLGA NPs show some disadvantages, such as initial burst, incomplete release, and limited surface functionalization. It is worth highlighting that PLGA exhibits some angiogenic properties that could interfere with the anticancer effects of the therapeutical molecules or, in the case of diabetic patients, accelerate wound healing [144].

### 6.4. Polyethylene Glycol

Polyethylene glycol (PEG) is an amphiphilic, nontoxic, biodegradable polymer that offers biocompatibility, stability, and prolonged blood circulation time for different drugs while promoting the accumulation at tumor sites, which results in the introduction of a therapeutic agent with a minimally invasive approach [145]. Thus, PEGylation or modification of biological molecules (including NPs) through covalent or noncovalent bonds with PEG represents a commonly used technique for improving the physicochemical properties of delivered agents [146]. PEG incorporation into an NP structure promotes water solubility of poorly soluble drugs, inhibits aggregation, decreases serum protein adsorption, and reduces capture rate by the reticuloendothelial system. However, PEG concentration is fundamental for determining the therapeutic effect of drugs because an increasing PEG concentration decreases the drug release rate that could increase the therapeutic effect and minimize the drug side effects. Moreover, PEG can diminish the initial burst that triggers an overmedication in conventional drug delivery systems [147].

Cationic nanocomplexes can be PEGylated for improving their stability by shielding their charge in order to prevent protein adhesion, aggregation with red blood cells, or activation of the immune system, which limits their potential clinical applications [148]. In the same way, polymer–lipid–PEG hybrid nanoparticle systems have emerged as a novel design for encapsulating anticancer drugs and photosensitizers to exhibit higher cytotoxicity in tumor tissues due to an increased drug loading and a reduced aggregation of the photosensitizer in aqueous solution, which promotes a faster cellular uptake [149]. PEG can be mixed with other polymers to prepare nanoparticles that reduce undesirable toxicity to healthy tissues and improve the pharmacokinetic properties of loaded anticancer molecules, such as PLA–PEG NPs [150].

Additionally, PEG can be modified to be pH-sensitive for promoting nanoparticle permeability in tumoral cells with the objective of decreasing noncancerous cellular uptake by releasing the radiosensitizer or chemotherapeutic drug inside the low-pH microenvironment of HNC cells [151]. Moreover, PEG can be conjugated with specific antibodies such as low-density lipoprotein receptor (LDLR) to target the tumor hypoxic region, which is the main contributor to chemoresistance. Thus, PEG could represent a useful nanocarrier for treating cancer by co-delivering a chemotherapeutic (cisplatin) and a chemosensitizer (metformin) into the hypoxia core area of tumors in a very promising strategy for treating HNC [152].

## 7. Stimuli-Responsive Drug Delivery Systems

**Figure 3:**
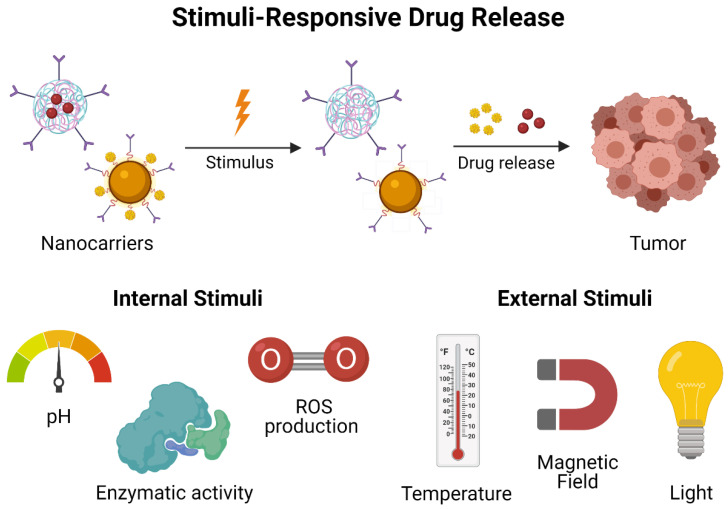
Schematic illustration of stimuli-responsive drug release. Drug release can be started by internal (pH, ROS production, and enzymatic activity) and external (temperature, magnetic field, and light) stimuli.

Intelligent biomaterials with stimuli-responsive properties have been studied as a novel generation of drug delivery systems for treating HNC in a more efficient way. Their mechanism of action is related to and somehow activated by the unique characteristics of the tumor microenvironment exhibited during cancer progression, such as low pH, enzyme overexpression, high levels of ROS, upregulation of antioxidants, and hypoxia. Therefore, smart materials focus on responding to these specific internal stimuli (Figure 3) such as changes in pH and enzymatic activity. In the same way, these materials respond to external stimuli (Figure 3), including exposure to light or the intensity of a magnetic field intensity, which leads to more accurate drug delivery, improved tumor penetration, and sustained drug release [153]. The functionalization of nanocarriers for increasing their bioavailability at tumor sites has been evolving for decades, until reaching the fourth generation of nanoformulations. The first generation was focused on passive drug release mechanism; the second generation implemented targeted and bioactive mechanisms of drug delivery, while the third and fourth generations search for guided assembly and intelligent biomaterials.

### 7.1. pH-Responsive Drug Delivery 

Tumor regions exhibit pH values between 5.6 and 7.0 in different sites, while the pH values in the bloodstream and healthy tissues are about 7.4. This difference may be attributed to the hypoxia conditions inside tumor cells, which promote the production of lactic acid, the activation of proteases, and the accumulation of metabolic waste [154]. Therefore, pH-responsive polymers have been designed to take advantage of the pH differences between tumors and normal tissues, highlighting that an acidic environment can decrease the activity of radio- and chemotherapy drugs, whereas it can be improved in thermotherapy agents [153].

On the other hand, pH-responsive polymers are classified in two main categories: (1) polymers with ionizable moieties and (2) polymers with acid-labile linkages. The first group of polymers is characterized by a reversible protonation and deprotonation of their ionizable groups depending on their pKa, leading to a sustained drug release. Meanwhile, the second group of polymers respond to pH changes by degradation through hydrolysis, which triggers the release of anticancer agents [155].

### 7.2. Enzymatic-Responsive Drug Delivery 

Cancer is characterized by enzymatic dysregulations and overexpression of certain enzymes, so a novel approach proposes the use of these enzymes as stimuli for intelligent and faster release of the drug at the tumor site, considering the exceptional selectivity of enzymes for their substrates [154]. The most studied enzymes for drug delivery in HCN are the matrix metalloproteinases (MMPs), which act as proteolytic enzymes by degrading the extracellular matrix proteins [156]. In addition, MMPs are implicated in the progression, migration, and metastasis of cancer. So, they have been considered as important biomarkers during cancer diagnostics, but they have also served as trigger stimuli for drug release [153].

### 7.3. ROS-Responsive Drug Delivery

An adequate balance in the levels of ROS is decisive in the cellular life of healthy tissues because ROS are related to proper control of cell proliferation and differentiation. For this reason, eukaryotic cells developed a complex scavenging system based on superoxide dismutases (SODs) located in the cytoplasm, mitochondria, and the extracellular matrix. In addition, cells contain glutathione peroxidases (GPXs), glutathione reductases (GRs), and catalases to convert superoxide anions in water and recycle the antioxidants in the reduced state [157]. However, many types of cancers exhibit mitochondrial dysfunctions, abnormal metabolic processes, and genetic mutations that cause high levels of ROS, up to 100 times higher than in normal cells. Thus, the researchers have been inspired in exploiting ROS as a tool for developing target-specific drug delivery systems for treating head and neck cancer [158].

ROS-responsive drug delivery systems have shown great potential by using molecules with ROS-cleavage groups such as thioether, thioketal, and organoborane compounds, which can be degraded through oxidation, leading to ROS-activated drug release [153,159].

### 7.4. Light-Responsive Drug Delivery

Photoresponsive targeting systems are useful for a temporary noninvasive controlled release of drugs in vivo due to their remote control. The most important element for achieving a light-response targeting is a chromophore with absorption peaks within ultraviolet, visible, or NIR regions. The chromophores are molecules that change their structural conformation by exposure to a specific light wavelength, allowing the release of the encapsulated drugs only when a certain light source is irradiated [154]. The light-responsive materials most used in photothermal therapies are those that act in the NIR region, since this type of light exhibits good penetration and minor damage to healthy tissues during HNC treatments [153]. However, NIR-mediated drug delivery must consider drug stability at relatively high temperatures, around 40–50 °C, to avoid alterations in drug activity. Otherwise, the efficacy of HNC treatments can be enhanced by combining the use of light-responsive drug delivery with the administration of photosensitizing molecules, which produce cytotoxic ROS capable of inducing apoptosis in tumor cells [160].

### 7.5. Magnetic-Responsive Drug Delivery

Magnetic nanoparticles (MNPs) are extensively applied in cancer treatments for increasing the accumulation of anticancer agents in tumors. The nanoparticles are guided by an external magnetic field that is manipulated remotely [46,74]. The most widely used MNPs are iron oxide nanoparticles, as already discussed in Section 3. Superparamagnetic iron oxide nanoparticles (SPIONs) have received regulatory approval for clinical use several years ago. These MNPs usually have a magnetic core with a polymer or silica coating to improve their biocompatibility and extend their half-life [161]. MNPs coating allows them to serve as vehicles for delivering antitumor agents in a controlled manner. Moreover, the surface of MNPs can be conjugated with specific targeting agents for dual active targeting to reduce the required dose of anticancer drugs and the side effects related to a systemic distribution [162]. Otherwise, MNPs tend to exhibit heat generation properties when an alternating magnetic field is applied. So, MNPs could serve also as hyperthermia agents, resulting in a synergic antitumor effect [153].

### 7.6. Temperature-Responsive Drug Delivery

The high rates of aerobic glycolysis in tumors and their fast proliferation result in an elevated temperature around 40–42 °C. So, researchers are studying the possibility of programming temperature-responsive nanocarriers based on the differences in temperature between tumor tissue and healthy tissues [154]. The most commonly used temperature-sensitive nanocarriers are liposomes, which are prepared by using phospholipids with transition temperature above the biological temperature (37 °C), thus allowing a controlled release of the entrapped drug after application of external hyperthermia [163]. Furthermore, temperature-sensitive liposomes exhibit rapid anticancer agent release while the tumor is heated, leading to an elevated intravascular drug concentration, around 3 to 25 times higher compared to available commercial delivery systems [164].

### 7.7. Immunotherapeutic Drug Delivery Systems

The ability to differentiate between pathological tissues and healthy tissues represents a substantial improvement in site-specific delivery of chemotherapeutic agents. Thus, new cancer treatments focus on the co-delivering of tumor-associated antigens (TAAs), neoantigens, and adjuvants to dendritic cells or on designing artificial antigen-presenting cells, both with the objective of reversing the immunosuppressive microenvironment in tumor tissues. The strategy is based on the fact that tumor cells tend to overexpress immune checkpoint molecules on their surface to inactivate T cells, mainly cytotoxic T cells (CD8+), which are responsible for killing abnormal, damaged, or infected cells. So, immunotherapeutic agents act as enhancers to stimulate T cell production and activation against cancer cells [49]. The most used antigens include anti-programmed cell death-1 and antitumor necrosis factor receptor superfamily member 4, which are related to enhanced activation of T cells in the tumor microenvironment, improved therapeutic efficacy, and an augmented generation of immunologic memory [165].

## 8. Other Nanoparticles

### 8.1. Mesoporous Silica Nanoparticles

Mesoporous silica nanoparticles (MSNPs) represent one of the most promising inorganic nanomaterials for cancer therapy. MSNPs possess several advantages, including uniform mesoporosity, tunable particle size (usually around 50–200 nm) and pore diameter (commonly between 2–5 nm, but larger up to 23 nm), versatile structure, easy surface functionalization, high surface area, large pore volume, high payload capability, biocompatibility, and biodegradability [166,167,168]. It is worth emphasizing that the customization of the pores is decisive for loading of hydrophobic or hydrophilic anticancer agents of different molecular weights, because the pore must be large enough to allow the physisorption or chemisorption of hydrophobic molecules from organic solvents and the electrostatic adsorption of hydrophilic molecules [169]. Silica has been classified as a “Generally Recognized as Safe” (GRAS) material by FDA. Moreover, the unique structure of mesoporous materials allows high drug loading capacity, high encapsulation efficiency, abundant possibilities for surface modification, and sustained drug release [168]. Thus, based on their unique properties, MSNPs could exhibit better results than other kinds of nanocarriers by overcoming drug resistance limitations and potentiating the anticancer effects of the treatments with reduced doses (compared with free drugs), while they diminish the toxicity and unfavorable side effects of chemotherapeutic drugs [170]. MSNPs can also co-deliver different anticancer agents to improve HNC treatment effects, as in the case of co-delivering a multidrug resistance protein 1 (MDR1) siRNA, to block the expression of MDR1 genes in cancer cells and prevent drug resistance, and doxorubicin, a chemotherapeutic drug for killing cancer cells. MSNPs can be coated with cationic polymers for the conjugation and delivery of DNA and siRNA molecules [166]. In addition, the coating could act as a gatekeeper system for a pH-dependent or redox-sensitive controlled drug release or as a half-life extender by hiding the nanoparticle from the mononuclear phagocytic system [168].

### 8.2. Solid Lipid Nanoparticles

Solid lipid nanoparticles (SLNPs) represent a new class of colloidal nanocarriers in the size range of 50–1000 nm. SLNPs contain a solid core made up of a high-melting-point lipid encapsulated by a monolayer of a safe surfactant [171]. SLNPs were developed as an alternative system to counteract several disadvantages of other lipidic systems (liposomes and micelles), including poor stability (phospholipid degradation), leakage of drug from the dosage form, the use of organic solvents during synthesis, sterilization problems, and production cost [172,173]. The improved performance of SLNPs compared with other lipidic systems is due to their specific differences, which are the composition (type of lipids used) and the structure of the nanocarriers (solid matrix vs. lipid layers) [174]. Thus, SLNPs could be used as a potential chemotherapeutic drug delivery system for enhancing the tumor-killing effect through an improved intracellular shuttling, hydrolytic stability of the anticancer drug, and drug leakage prevention during systemic administration [175]. In addition, SLNPs exhibit low toxicity as their lipid core is composed of physiological biodegradable lipids that provide better protection to loaded drugs against chemical degradation, thus improving the bioavailability of the anticancer molecules with a reduced risk of developing chronic and acute toxicity. SLNPs can encapsulate hydrophilic and lipophilic drugs with an enhanced drug encapsulation efficiency compared with other nanoparticle formulations [176]. However, SLNPs may exhibit poor drug-loading capacity and a limited drug expulsion due to polymorphic transition during storage, which can be overcome by modifying SLNP structure through the association of liquid lipids to the solid structure. The liquid lipids produce some imperfections in the solid matrix to allow an increased drug concentration to be loaded and prevent an early burst delivery of drugs [177]. For instance, a novel approach of cancer treatments with phytochemicals, such as andrographolide (ADG), is emerging as a promising strategy to delay carcinogenesis in HNC. However, these natural compounds are highly hydrophobic and poorly bioavailable. So, SLNPs represent an ideal nanovehicle for countering ADG drawbacks, leading to a more effective encapsulation of hydrophobic compounds with an enhanced cellular uptake, which is related to an improved anticancer activity at a low dose compared with free drug, during oral administration [178].

A summary of the different nano-systems for HNC treatment described in the previous sections is provided in Table 1.

## 9. Diagnosis of Head and Neck Cancers (HNCs)

### 9.1. Traditional Approaches for Diagnosis of HNC

Head and neck cancer (HNC) is a category of cancers that typically begin in the throat (pharynx), mouth, nasal cavity, voice box (larynx), salivary glands, and sinuses, which are lined with squamous cells. HNC is more likely to occur in people over 50 years of age and twice as likely to occur in males [179,180]. Traditional diagnosis approaches for HNC are nasopharyngolaryngoscopy, head MRI, CT of the sinuses, CT of the head, panoramic dental X-ray, PET/CT, and chest imaging [181,182]. Despite the wide use of the above strategies in HNC diagnosis, low effectiveness requires researchers to find more efficient approaches. Advanced techniques of medical imaging and treatment are currently being carried out by nanotechnology. The growth of nanoscale imaging has the potential to boost the field of medicine by providing more accurate pictures of cellular processes. Existing imaging techniques are being adapted to improve their ability by using nanoscale materials and contrast nanoagents.

### 9.2. Emerging Biomarkers in Head and Neck Cancer in the Era of Targeted Diagnosis

Head and neck cancer (HNC) biomarkers have been evolving rapidly in recent years. As possibly robust biomarkers in HNC, many significant tumor tissue biomarkers with adequate clinical validation have been reported. Among the key prognostic markers for survival, cyclin D1 (CCND1), p16, epidermal growth factor receptor (EGFR), B-cell lymphoma extra-large (Bcl-xL)/Bcl-2, ERCC1, human papillomavirus (HPV), and the upregulation of genes such as EMS1, CCND, and FGFR1, have confirmed some indications in clinical trials [183]. The most significant and confirmed biomarkers in HNC are p16, interleukin-8 (IL8), and human papillomavirus. The detection of these low-concentration biomarkers is a difficult area in the field of diagnosis. Because of the characteristic features of nanomaterials that distinguish them from bulk materials, nanomaterial-based sensors have many advantages in accuracy and precision over sensors made from conventional materials. Nanosensors usually show enhanced sensitivity because they act on a scale comparable to natural biological systems and can interact more effectively with recognition elements of biomolecules [184].

### 9.3. Nanomaterials for the Imaging of Head and Neck Cancer

Early diagnosis of head and neck cancer (HNC) is a significant clinical concern and, due to a lack of signs, just one-third of HNC patients are diagnosed at an early stage [185]. Functional nanomaterials are appearing as versatile systems in nanomedicine, particularly in the field of biomedical imaging and treatment [186,187,188]. Various surface chemistries, peculiar magnetic properties, tunable excitation and fluorescence properties, and recent developments in the design and synthesis of different nanoparticles indicate their high potential [189,190,191]. Current medical imaging techniques (MRI, CT, US, etc.) are being updated to improve their ability by using nanoscale materials and contrast agents (see Table 2) [192].

Many authors have reported the use of nanomaterials for improved imaging diagnosis. For example, Kumar et al. reported the preparation and characterization of phospholipid nanomicelles for integrated MRI and NIR optical imaging of HNC. Nanomicelles were encapsulated by Pt(II)-tetraphenyl-tetranaphthoporphyrin (Pt(TPNP)) as near-infrared (NIR) phosphorescent molecules that were modified with gadolinium (Pt(TPNP)-Gd) [193]. Maximum intensity projections produced from 3D T1-weighted pictures showed an increased contrast for tumor, liver, and blood vessels. The broad spectral distinction between the absorption of Pt(TPNP) (~700 nm) and the emission of phosphorescence (~900 nm) resulted in a significant reduction in the background level (noise), resulting in great-contrast optical (NIR phosphorescence) imaging.

In another study, Colombé et al. prepared and tested gold nanoclusters (Au NCs) covered by zwitterionic or pegylated ligands with the purpose of evaluating the ability of Au NCs for optical image-guided surgery of HNC [194].

The development of a noninvasive and targeted system for early detection of oral squamous cell carcinoma (OSCC) is a grand challenge in cancer diagnosis. In this context, Li et al. prepared graphene oxide (GO) coupled with AF750-6Ahx-Sta-BBN (GO-BBN-AF750) GRPR-specific peptides and explored their cell uptake, receptor binding, and internalization in HSC-3 cells for early OSCC diagnosis [195]. A similar binding affinity to GRPR on HSC-3 cells was observed for GO-BBN-AF750 and AF750-6Ahx-Sta-BBN. GO-BBN-AF750 displayed cellular internalization properties in comparison to the AF750-6Ahx-Sta-BBN antagonist peptide.

Repeated doses of ^188^Re liposome were injected into the orthotopic tumor model by Chang et al. to examine the pharmaceutical and biological reactions of the high ^188^Re liposomal concentration injected in the buccal regions of nude mice [196]. Cerenkov luminescence imaging (CLI) was conducted to display the increased accumulation of ^188^Re liposomes after multiple administrations as compared to a single dose. Findings demonstrated that radiopharmaceutical PEGylated liposome-encapsulated rhenium-188 enhanced nanocarrier aggregation and inhibited human HNC cell proliferation.

The production of strong, nontoxic, tumor-targeted near-infrared (NIR) probes as contrast agents is important for the successful intraoperative detection of cancers [197]. By offering strong and photostable fluorescent probes, luminescent semiconductor quantum dots (QDs) have many distinct benefits for in vivo cell imaging [198]. In order to try this, Yakavets et al. prepared NIR-emitting ZnCuInSe/ZnS core/shell QDs (~750 nm) and connected them to A20FMDV2 peptide to address αvβ6 integrin-rich HNC [199]. In the 2D monolayer and 3D spheroid in vitro HNC models, QD-A20 displayed alpha-β6 integrin-specific binding. Thus, QD-A20 can be considered to be a highly effective nanoprobe for NIR biosensing and imaging-guided surgery.

The design of 3D neoplasm models in the preclinical research workflow is attractive in promoting the progress of therapeutics in clinical trials and in reducing the number of animals needed for in vivo models [200]. A powerful and high-throughput hanging drop technique for the development of 3D models of both HPV-positive and HPV-negative HNC has been reported by Santi et al. [201].

In order to attain a better antitumor effect for HNC, Wang et al. prepared a nano-hydrogel that responded to doxorubicin–indocyanine green matrix metalloproteinase (NDIMH) [202]. NDIMH demonstrated perfect characteristics of photosensitivity to light. With an 808 nm near-infrared (NIR) irradiation, NDIMH successfully blocked the proliferation, colonization, and metastasis of SCC-15. The hydrogels demonstrated a favorable synergistic antitumor effect and appropriate biosafety following intratumoral injection of NDIMH and 808 nm NIR illumination. In addition, fluorescence imaging revealed that NDIMH could increase the preservation of nanodrugs at the tumor cells dramatically.

An innovative AuS-based multimodal MR/SERRS device for imaging of HNC was developed by Sun et al. [203]. The ability to classify both tumor xenograft and metastatic lymph nodes by MRI preoperatively and by SERRS intraoperatively not only eliminates the need for excessive resection of neurological structures but also offers a new chance to boost the surgical prognosis of infiltrative HNC.

### 9.4. Nanosensors in Diagnosis of Head and Neck Cancer 

Nanosensors may enhance specificity because they act on a scale comparable to natural biological systems, enabling chemical and biological molecules to function with detection events that trigger observable physiological changes [204].

**Figure 4:**
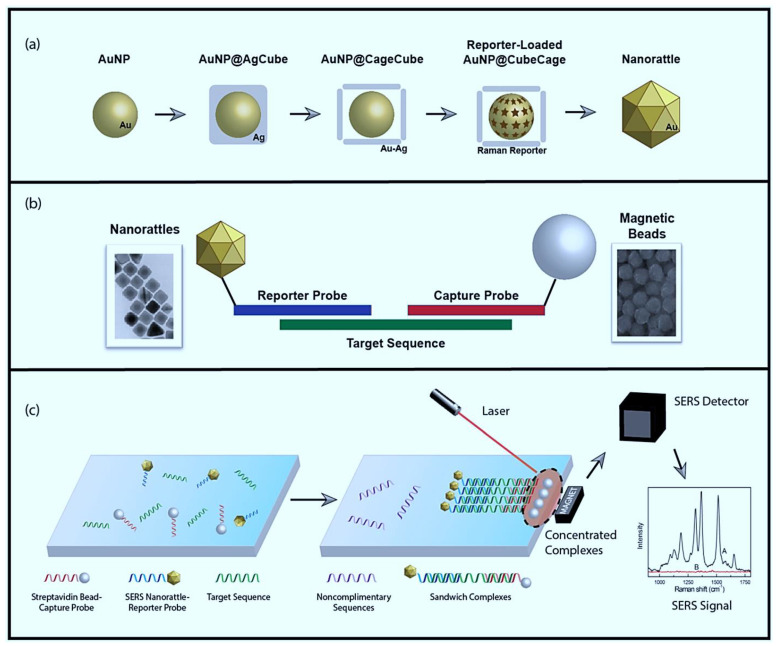
SERS diagnostic process summary. (**a**) Synthesis of cubic nanorattles, beginning with single-crystal, spherical gold nanoparticle (AuNP) cores. The AuNP cores are coated with cubic Ag shells to obtain AuNP@AgCube. Galvanic replacement transforms cubic Ag shells into cubic Au–Ag cages containing AuNP in the interior. Raman reporters are loaded, and the porous cubic cages are turned into complete shells by a final Au coating to obtain cube nanorattles. (**b**) Structure of individual hybridization complex. Gold nanorattles (Au-NR) are functionalized with DNA reporter probes, and streptavidin beads are functionalized with DNA capture probes. Both probes are complimentary to the specific cytokeratin sequence. (**c**) Extracted nucleic acids are incubated with functionalized nanorattles and beads, then washed away. Remaining complexes undergoing successful hybridization are isolated using a strong magnet and concentrate to a spot for laser excitation in order to yield a SERS signal. Reproduced from [205].

Vohra et al. prepared Au nanorattles with a surface-enhanced Raman scattering (SERS) feature that targeted specific biomarkers of HNC (cytokeratin nucleic acid) (Figure 4) [205]. Their method showed 89% specificity and 100% sensitivity and was therefore proposed as a valuable alternative to histological examination.

Wang et al. prepared an electro-chemiluminescence nanosensor (ECL) based on nano-polythionine (NPTh) core–shell nanocomposites for early diagnosis of HNC by detection of the p16INK4a gene [206]. The intensity of ECL on the NPTh electrode changed linearly by logarithm of the p16INK4a gene concentrations in a wide range, with an LOD of 0.05 pM and a signal/noise ratio of 3.

**Figure 5:**
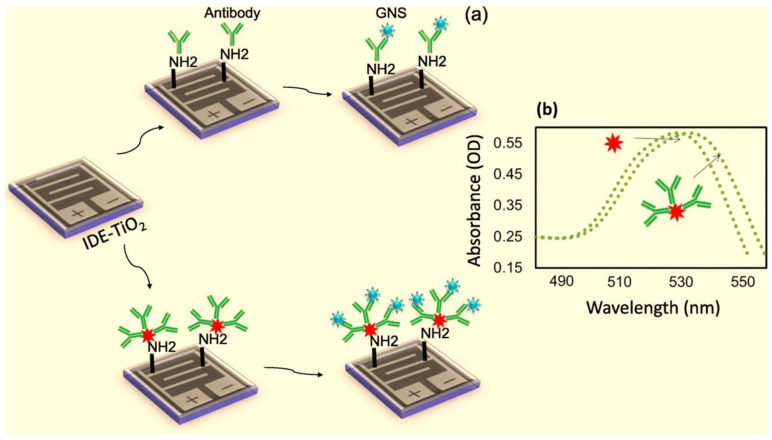
(**a)** Schematic representation for the detection of SCC-Ag. IDE-TiO2 surface was modified into amine by APTES followed by the immobilization of antibody or GNS antibody. The amine group from APTES reacted with the carboxyl group on the antibody. SCC-Ag was detected by the interaction at the antigenic region and compared. (**b**) UV-Vis spectroscopy measurements with GNS. Scanning was in the region between 480 and 560 nm, and the peak maxima were ~530 nm. The arrows indicate GNS with and without antibody. Reproduced from [208].

Squamous cell carcinoma antigen (SCC-Ag) is a tumor biomarker and has been shown to have elevated levels in patients with HNC and is strongly associated with tumor volume [207]. The amount of SCC-Ag level on a modified titanium oxide (TiO_2_) electrode was detected and quantified successfully by Wang et al. (Figure 5) [208]. The LOD of SCC-Ag was 100 fM, while it was improved to 10 fM when the antibody was gold nanostar-attached, reflecting a 10 fold increase. Interestingly, this sensitivity increase was found to be 1000-fold greater than that of other substrates.

A nanosensor based on multifunctionalized Au NPs was developed by Yokchom et al. for detection of p16INK4a [209]. Clinical assessments showed a precision of 85.2% with a 0.69 kappa coefficient.

Nanomotors are engineered nanomaterials that can be driven and functionalized by various mechanisms for particular applications, such as biosensors [210]. Qualliotine et al. developed a nanomotor based on ultrasound-assisted Au nanowire for detection of HPV16 E6 mRNA transcripts. Nanowires were functionalized with dye-labeled single-stranded DNA and graphene oxide [211]. Results showed 60.7% of maximum fluorescence recovery obtained in nanomotors incubated with RNA (extracted from HPV-positive OPC cells), whereas incubation with RNA extracted from HPV-negative cells generated zero fluorescence. Minimum fluorescence (0.01 au) was provided by nanomotor incubation with intact HPV-negative cells, while incubation with HPV-positive cells produced a visible signal (0.43 au) under static conditions and had 2.3 times higher strength when driven by ultrasound.

Soares et al. reported a low-cost genosensor capable of detecting low HPV16 concentrations in buffer samples and separating cell lines of HNC according to their HPV16 status with high precision [212]. This genosensor comprised a microfluidic system mounted on a layer-by-layer film of chitosan and chondroitin sulfate that had an active layer of an HPV16 capture DNA probe (cpHPV16). Obtained LOD for complementary ssDNA HPV16 oligos (ssHPV16) was 10.5 pM. The genosensor was also able to differentiate between HPV16+ and HPV16- cell lines using the interactive document mapping multidimensional projection approach.

In a similar study, in patients with HPV16-positive head and neck cancer, Farzin et al. reported an amine–ionic liquid functionalized reduced graphene oxide (NH_2_-IL-rGO) nanosensor for electrochemical human papillomavirus (HPV16) DNA detection in HNC [213]. The developed genosensor was capable of detecting ultralow HPV16 DNA concentrations with an LOD of 1.3 nM (3σ) and a linear range of 8.5 nM to 10.7 μM. Unlike other works which used PCR products, the extracted clinical sample DNA was used on the pDNA-modified electrode to confirm the effectiveness of the process.

Zhu et al. developed a new approach based on spermine-modified nanodiamonds (SP-NDs) to selectively enrich oligonucleotides of human papillomavirus virus (HPV) [214]. Findings showed that SP-NDs from sodium dodecyl sulfonate and urea solution would successfully isolate DNA oligonucleotides. SP-NDs can also preferentially extract oligonucleotides from enzymatic digestion products. Thus, without any further purification, the extract can be detected directly, and according to the mass (MS) results, the genotyping of HPV can be accomplished. More specifically, clinical samples contaminated with HPV genotypes 16 and 18 can be detected with SP-ND extraction.

## 10. Conclusions

A common cancer that affects the areas of the mouth, throat, and salivary glands is head and neck cancer (HNC). The diagnosis of HNC with an effective biomarker and imaging approach is necessary in order to detect cancer at an early stage and improve the patient’s outcome and quality of life. The most significant and confirmed biomarkers in HNC are p16 and human papillomavirus status. Despite the wide use of the above methods in HNC diagnostics, low effectiveness has encouraged researchers to find more efficient alternative methods. Nanomedicine is currently being used in groundbreaking medical imaging techniques and biomarker detection. The growth of nanoscale imaging has the potential to boost the field of medicine by providing more accurate pictures of cellular processes. In the same way, HNC treatments present poor results due to several limitations mainly related to conventional delivery methods for anticancer agents. Thus, different drug delivery approaches based on nanotechnology have been developed to enhance the potential of therapeutical drugs through a targeted strategy and improve bioavailability at tumor sites, as we summarized throughout the article. In addition, nanodelivery devices allow the co-delivery of anticancer agents for countering tumor resistance. A promising strategy in anticancer nanomedicine relies on using stimuli-responsive drug delivery systems that potentiate drug therapy efficacy and localization while reducing side effects. However, nanotechnology still has a long way to go for extensive use in clinical applications.

## Figures and Tables

**Table 1:** Comparison between the properties of the nanoparticle systems most used for the treatment of head and neck cancer.

Nanoparticle System	Material Characteristics	References
Metallic and metal oxide nanoparticles	Metallic NPs are solid inorganic particles (among 1–100 nm) that are commonly used in HNC treatments based on their physicochemical properties: - Biocompatibility - Photostability - Photothermal conversion - Optical features - Radiosensitizer activity - Surface functionalization versatility - Enhanced permeability and retention effect - Plasmon resonance properties (Au) - Photocytotoxic effects (Gd) - Magnetic properties (Fe_3_O_4_) - Cellular cytotoxicity induction (Ag) - Redox-modulatory effect (CeO_2_) - Easily recognized by innate immune system - Long-term health risk still unknown	[46,50,51,54,59,66,68,69,74,77,84]
Liposomes	Liposomes are artificial nanometric (around 20–150 nm) vesicles formed by a phospholipid bilayer with an aqueous inner space. They are widely investigated as a nanocarrier for anticancer agents, based on their properties: - Biodegradability - Biocompatibility of the phospholipid bilayer - Entrapment of hydrophilic and hydrophobic molecules (individually or simultaneously in the aqueous cavity) - Affinity to mammalian cell membranes - Enhanced cellular uptake and biodistribution - High drug payload - Simple synthesis methods - Low batch-to-batch variability - Easy surface conjugation - Formulation flexibility	[89,90,91,95,96,97]
Nanomicelles and microemulsions	Nanomicelles are colloidal nanoparticles (about 5–100 nm) synthesized from amphiphilic monomers that self-aggregate. They consist of two main regions: an inner hydrophobic core and an outer hydrophilic shell. Nanomicelles exhibit interesting drug delivery features based on their characteristics: - Encapsulation of nonpolar molecules (hydrophobic core) - Easy surface conjugation with polar drugs or ligands - Enhanced solubility of hydrophobic molecules - Drug targeting and specificity - High drug load capacity - Great colloidal stability - Stimuli-responsive (pH, temperature, light, ultrasound) - Longer circulation time (avoid the reticuloendothelial system) - Co-delivery of hydrophobic and hydrophilic anticancer agents - Premature drug release - Poor control of sustained release - Inability to encapsulate hydrophilic agents	[105,106,108,114]
Polymeric nanoparticles	Polymeric nanoparticles are colloidal particles (among 100–300 nm) prepared with biocompatible polymers for drug delivery. They offer several advantages during drug delivery, based on their properties: - Biodegradability - Biocompatibility - Natural or synthetic polymers - Predictable pharmacokinetics (synthetic polymers) - High encapsulation efficiency - Controlled drug release - Cationic, anionic, or nonionic properties (depending on the polymer) - Mucoadhesive features (chitosan) - pH sensitivity (chitosan) - Initial boost prevention - High purity and reproducibility (synthetic polymers) - Nonimmunogenic properties (natural polymers) - Coating features - Enhanced solubility - Easy preparation technique - Good control over size and size distribution - Prolonged blood circulation (synthetic polymers) - Approved by FDA and EMA (PLGA) - Reduced reticuloendothelial system captures - Poor stability and easy degradation (natural polymers) - Possible accumulation in the liver or the spleen	[54,106,117,118,119,120,127,128,133,134,139,145]
Mesoporous	Mesoporous silica nanoparticles represent a promising inorganic nanocarrier (among 50–200 nm) with very interesting properties, such as: - Biocompatibility - Biodegradability - Tunable pore size - Easy surface functionalization - Large mesopore volume - Uniform mesoporosity - Flexible structure - High payload capability - Great encapsulation efficiency - Sustained drug release - Minimum toxicity - Electrostatic adsorption of hydrophilic molecules - FDA approval	[166,167,168,169]
Solid lipid nanoparticles	Solid lipid nanoparticles are colloidal nanovehicles (around 50–1000 nm) synthesized with lipids that remain solid at room temperature and surfactants. Among their main characteristics are: - Biocompatibility - Biodegradability - High drug stability - Improved intracellular uptake - Prevention of drug leaking during administration and prevention of an early burst delivery - Feasibility of incorporating hydrophilic and lipophilic molecules - Improved bioavailability of poorly water-soluble agents - Possible drug expulsion during storage - Poor drug loading capacity	[171,172,173,175,176,177,178]

**Table 2:** Main characteristics of the different nanoparticle systems most used for the imaging of head and neck cancer.

Nanomaterial	Main Characteristics	Refs.
Phospholipid structures	Increased contrast for tumor, liver, and blood vessels Broad spectral distinction between the absorption of Pt(TPNP) (~700 nm) Enhanced nanocarrier aggregation	[193,196]
Gold NPs	Boost the surgical prognosis of infiltrative HNC Optical image-guided surgery of HNC	[194,203]
Graphene	Similar binding affinity to GRPR on HSC-3 cells Cellular internalization properties	[195]
Quantum dots	Alpha-β6 integrin-specific binding Highly effective nanoprobes for NIR biosensing and imaging-guided surgery	[198]
Hydrogel	Favorable synergistic antitumor effect and appropriate biosafety Increase the preservation of nanodrugs at the tumor cells	[202]

## Data Availability

Data sharing not available.
